# D-Amino Acids and D-Amino Acid-Containing Peptides: Potential Disease Biomarkers and Therapeutic Targets?

**DOI:** 10.3390/biom11111716

**Published:** 2021-11-18

**Authors:** Mohamed Abdulbagi, Liya Wang, Orwa Siddig, Bin Di, Bo Li

**Affiliations:** 1 Department of Pharmaceutical Analysis, China Pharmaceutical University, Nanjing 210009, China; mohamedabdulbagi@gmail.com (M.A.); leah_w0503@163.com (L.W.); orwasiddig@gmail.com (O.S.); 2 Center Key Laboratory on Protein Chemistry and Structural Biology, China Pharmaceutical University, Nanjing 210009, China; 3 MOE Key Laboratory of Drug Quality Control and Pharmacovigilance, China Pharmaceutical University, Nanjing 210009, China

**Keywords:** D-amino acids, DAACPs, biomarker, therapeutic targets, LC-MS, D-Asp, D-Ser

## Abstract

In nature, amino acids are found in two forms, L and D enantiomers, except for glycine which does not have a chiral center. The change of one form to the other will lead to a change in the primary structure of proteins and hence may affect the function and biological activity of proteins. Indeed, several D-amino acid-containing peptides (DAACPs) were isolated from patients with cataracts, Alzheimer’s and other diseases. Additionally, significant levels of free D-amino acids were found in several diseases, reflecting the disease conditions. Studying the molecular mechanisms of the DAACPs formation and the alteration in D-amino acids metabolism will certainly assist in understanding these diseases and finding new biomarkers and drug targets. In this review, the presence of DAACPs and free D-amino acids and their links with disease development and progress are summarized. Similarly, we highlight some recent advances in analytical techniques that led to improvement in the discovery and analysis of DAACPs and D-amino acids.

## 1. Introduction

Amino acids, the building blocks of peptides and proteins, are found naturally in two forms (L and D enantiomers), except for glycine which does not have a chiral center. L and D enantiomers have different orientation of the four substituents attached to the α-carbon and known as non-superimposed mirror images. The tertiary structure of proteins is critical for their activity, therefore a change in one amino acid stereoisomerism may lead to dramatical change in their physicochemical properties and activity. It was a long-lasting belief that only the L-forms are incorporated in the synthesis of proteins, and this homochirality of L-amino acids in protein synthesis was one of the mysteries in life. Nevertheless, in living organisms, not only free forms of D-amino acids, but also several D-amino acid-containing peptides (DAACPs) have been isolated [1,2]. Among these DAACPs many are biologically active while their counterpart peptides synthesized from only L-amino acids are either totally inactive or have minimum activity [3]. The incorporation of D-amino acids in proteins may result from different mechanisms; non-enzymatic racemization linked with ageing or diseases or by enzymatic post-transnational modification (PTM) for the mRNA-encoded L-amino acids [2].

## 2. Sources of D-Amino Acids

### 2.1. Enzymatic Conversion of L-Amino Acids

As L-amino acids are present more predominantly in living organisms, they act as a substrate for D-amino acid synthesis. This conversion reaction occurs in the presence of the enzyme racemase, which changes the stereochemistry of the chiral α-carbon in these amino acids [4]. Several racemase enzymes have been studied in different organisms, such as alanine racemase which catalyzes the generation of D-Ala, a crucial component of the peptidoglycans, from L-Ala [5,6]. Likewise, glutamate racemase is responsible for the conversion of L-Glu to D-Glu [7]. The presence of these D-isomers is thought to protect the bacteria from the attack of different peptidases [8]. In mammals, serine racemase is the enzyme that inverts the stereochemistry of the α-carbon of L-serine to produce D-serine [9]. Notably, D-Ser, albeit found in low amounts compared to L-Ser, is a potent co-agonist of the N-methyl-D-aspartate (NMDA) glutamate receptor. Moreover, D-Asp and D-Ala are also found in the mammalian brain, and though found in lower amounts than D-serine they are still detectable [10,11]. Additionally, bacteria and other species also synthesized a group of compounds, known as antimicrobial peptides or host defense peptides, for their natural immunity and existence [12]. Since their discovery they have been viewed as an alternative to conventional antibiotics and several are already in clinical use, such as Gramicidin S and Bacitracin. Among the distinctive features of these peptides is the presence of D-amino acids in their structures which would make them more resistant to proteolysis [13]. Furthermore, the incorporation of D-amino acids in some peptides led to improvements in the antibacterial action [14,15,16,17]. Nevertheless, the excessive modification of L-amino acids with their D-form counterparts in the structure of the biologically active peptides is linked with potential risks of cytotoxicity and immunogenicity [13]. In addition to antibacterial peptides, there are other drug molecules that contain D-amino acids in their chemical structure, such as the antidiabetic nateglinide and the thrombin inhibitor PPACK [18,19]. The growing interest in the chemical and pharmaceutical industries for pure amino acid enantiomers necessitates establishing an efficient reaction system for their synthesis [20]. Accordingly, different L-amino acid deaminases and D-amino acid oxidases were extensively studied to obtain pure amino acid enantiomers [21,22,23,24]. The reader is strongly recommended to refer to Molla et al. and Nakano et al. for more comprehensive information in this point [20,25].

### 2.2. Spontaneous Inversion of Isomerism

**Figure 1:**
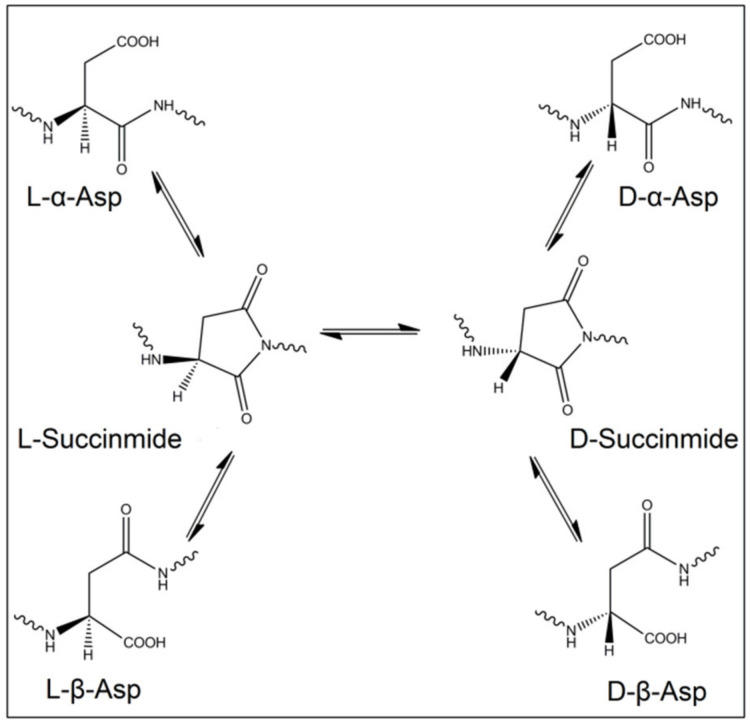
Scheme of the spontaneous isomerization of the four isomers of Asp through the succinimide intermediate.

Importantly, not only racemases are responsible for the inversion of L-amino acids into the D-form, but this process can occur spontaneously, despite being very slow, in long-lived proteins and tissues. The reasons behind these changes in stereoisomerism are of non-enzymatical origin, instead, they occur due to chemical reactions that resulted in inversion of the amino acid chirality. Indeed, oxidative stress and free radicals were proved to induce such modifications in proteins and peptides [26,27]. Even though these reactions are very slow in kinetics, nevertheless, they are valuable as a mirror to reflect the age of these proteins and tissues. Among the 19 chiral proteinogenic amino acids, L-Asp is the most exposed to racemization. The inversion of the isomerism of L-Asp mainly happens through a succinimide intermediate. L-succinimide can be formed when the lone pair of electrons of the amino acid adjacent to L-Asp attack the carboxyl group of L-Asp. Then, the formed L-succinimide can be converted to D-succinimide by keto-enol tautomerism. Both L/D-succinimide can be hydrolyzed to form four stereoisomers, L-α-Asp, L-β-Asp, D-α-Asp and D-β-Asp (Figure 1). Additionally, L-Asn deamidation will result in the same four isomers of Asp [28]. It is known that L-Asp and L-Asn can be isomerized to D-form when the following amino acid has a small side chain, such as Ala, Gly and Ser.

### 2.3. Food

Food and dietary products, both natural and processed, contain different amounts of D-amino acids. In fact, several D-amino acids are present in fruits and vegetables such as apples, grapes and tomatoes [5]. Unlike human milk, the milk of cows, goats and ewes contain free D-Ala, D-Asp, D-Glu and D-Ser [5,29]. There have been numerous observations that food processing resulted in stereoisomerism inversion of the proteinogenic L-amino acids and the production of the D-counterparts. Accordingly, many researchers studied the effects of different conditions in food making processes. Several manufacturing processes are involved in making foods, such as high temperatures, fermentation, strong acid and alkali treatments. Ultimately, this all together resulted in increase in the D-amino acids formation [30]. Of worth noting is that many D-amino acids have a more sweet taste than the L-enantiomers, for example the dipeptide Alitame which contains L-Asp and D-Ala is 200 times sweeter than sucrose [31]. Not surprisingly, this led to the increased use of them as an alternative to sucrose.

### 2.4. Microbiota

Another source for the D-amino acids in mammals is microbiota that present in the gut. As mentioned earlier, D-amino acids are important constituents of the peptidoglycans in the bacterial cell wall. The two most common D-amino acids that are found in bacterial cell walls are D-Ala and D-Glu [8]. Nonetheless, in some other bacterial cell walls more D-amino acids have their position in peptidoglycans synthesis, such as D-Ser and D-Asp [32].

## 3. Catabolism of D-Amino Acids

Interestingly, the presence of the D-amino acid oxidase (DAO) enzyme corrects the amounts of D-amino acids by converting them to imino acid by oxidative deamidation. Although DAO was firstly discovered almost one hundred years ago, its biological importance is only recently well understood, and only after the discovery of D-Ser in human brain [33,34]. Likewise, another enzyme that is accountable for D-Asp degradation, D-aspartate oxidase (DDO), was discovered some time ago. DDO is a peroxisomal flavoenzyme that oxidizes D-Asp in presence of H_2_O and O_2_, producing oxaloacetate, H_2_O_2_, and NH_4_^+^. Notably, this enzyme cannot degrade D-Ser [6,35]. DAO and DDO control the levels of D-amino acids in the body to certain levels.

Despite the presence of these enzymes, it bears mentioning that significant increase in the levels of D-amino acids have been found in several disease conditions. Additionally, several DAACPs have been isolated from different tissues in several diseases [36,37,38,39]. The significant increase in certain D-amino acids together with the growth in the discovered DAACPs in these diseases surge the consideration of them as potential biomarkers [40,41,42,43,44,45]. Unveiling the molecular mechanisms behind the formation of DAACPs and the alteration in D-amino acid metabolism will inevitably assist in understanding these diseases and finding new biomarkers and drug targets. This is particularly the case with the recent advances in the analytical techniques that allow for the detection of very small amounts of analytes, and are able to accurately distinguish several compounds having very similar chemistry. Consequently, this will accelerate the disease discovery and/or finding of new treatments. In this review, we summarize the recent identification and findings of different D-amino acids and DAACPs from several diseases, and their possible role in the disease development and progress. In addition, we highlight the recent advancements in analytical techniques that will eventually lead the way to more discovery of new DAACPs.

## 4. D-Amino Acids and DAACPs in Selected Diseases

### 4.1. Alzheimer’s Disease

D-Asp and D-Ser have been found in brain tissues isolated from patients with Alzheimer’s disease (AD) [2]. This disease is linked with oxidative stress, a situation that arises from the presence of reactive oxygen species (ROS) [46]. AD is as an age-related neurodegenerative disease, and it is the most common cause of dementia in the elderly. Histopathological hallmarks of AD are intracellular neurofibrillary tangles and extracellular formation of senile plaques composed of the amyloid-beta peptide (Aβ) in aggregated form along with metal-ions such as copper and other metals. Copper can catalyze the production of ROS, such as hydroxyl radical, when bound to the Aβ. The produced hydroxyl radical and other ROS may result in the oxidative damage on both the Aβ peptide itself and other proteins in the brain. Another hallmark of AD is the presence of intracellular neurofibrillary tangles, which are composed of hyperphosphorylated Tau protein [47,48,49]. The Aβ has 38 to 42 amino acids, and this peptide is formed from the amyloid β-protein precursor by the sequential cutting actions of β- and γ-secretases [50]. The truncated and toxic fragment Aβ 25–35/40 which contains D-Ser at position 26 is believed to damage neurons and may account for the neurodegeneration in AD [51]. Moreover, D-serine levels in both blood and cerebrospinal fluid are found significantly higher in AD patients; as a result D-serine is believed to be responsible for controlling the extent of NMDAR-mediated neurotoxic changes that lead to AD [52,53,54,55]. However, these findings are contradicted by other researchers and further studies are therefore needed [56]. A recently published study suggested elevated levels of D-Ser could predict worse symptoms of memory decline, in particular the domains for word recall task, orientation, comprehensive and word-finding difficulty [57]. The reader is encouraged to refer to an excellent review for the roles of D-Ser in brain [58]. D-Asp also found its place in the investigation of the possible roles of D-amino acids in the brain and the links with some CNS dysfunctions. Free D-Asp could have a role in the development and maturation of tissues and organs in mammals. Indeed, D-Asp is found in the endocrine glands and its levels increase after birth as the maturation of these organs proceed. On the contrary, in the brain, free D-Asp amounts strongly decline following birth, and this is recognized as a consequence of the postnatal of DDO. In the brain, D-Asp is found mostly concentrated in the synaptic vesicles of terminal axon, thus proposing its function as an endogenous neurotransmitter [59,60]. A significant decrease in the amounts of D-Asp has been identified in the prefrontal cortex of patients with schizophrenia, this was accompanied with an elevated expression of DDO mRNA [61]. As highlighted earlier, incorporation of D-amino acid (such as D-Asp) in proteins produces different side chain orientation. Eventually, this results in changes in the secondary and tertiary structure, as well as the quaternary arrangement of a protein, which leads to dysfunctional proteins [62,63]. Briefly, Asp isomerization might lead to abnormal aggregation and degradation and induce partial protein unfolding, such as the Aβ aggregations that characterizes AD. Certainly, the racemization of Asp at position 23 accelerated the peptide aggregation and fibril formation [64]. Of final note is that there are many neuropeptides isolated from different organisms that contain D-isomer in their chains and the discovered number is rising. More importantly, theses neuropeptides have potent biological activities compared to those with all L-amino acids [65,66]. Dermorphin represents an excellent example of such neuropeptides. This heptapeptide has a D-Ala in the second position and is superior to morphine [67].

### 4.2. Cataract

Different PTMs have been characterized in the proteins of human eyes, include phosphorylation, Cys and Met oxidation, methylation and racemization. The latter is thought to be more closely related to the development of cataracts in eyes compared to others [68]. Crystallins are important structural and functional proteins in the human lens, and they have pivotal role in lens transparency. In the lens there are three types of crystallins, assigned as α-, β-, and γ-crystallins. For the lens to remain transparent, these proteins should retain their structures, as accumulative precipitation of lens proteins eventually will result in cataracts [69,70]. Several D-amino acids have been identified in human lenses including D-Asp, D-Ser, D-Glu/Gln and D-Phe. Moreover, the amount of the racemization to D-Ser and D-Asp was significantly higher in cataract lenses than in age-matched normal lenses. Indeed, cataract lens proteins from patients at 40 years old showed comparable racemization of Asp as lenses of 80-year-old normal people [69]. Not surprisingly, Asp racemization in lenses is the most studied among the others as it is the most susceptible amino acid for inversion in chirality. Asp racemization was identified in the two subunits of α-crystallins, named αA-crystallins and αB-crystallins, and such modification in amino acids chirality might disrupt the polymeric state of α-crystallins. In αA-crystallins, different Asp positions were found isomerized including Asp58, Asp84 and Asp151. Unavoidably, this results in decreased solubility and impairs the function of this important component of the lens proteins, contributing to the development of cataracts [68,71,72]. Likewise, in another study, Asp4 and Asp96 of αB-crystallin and Asp37 of βA3-crystallin were found isomerized to the D-form. These findings may also specify the possibility that the inversion of Asp residues could lead to the dissociation of αB- and βA3-crystallins from the polymeric and oligomeric states [70,73]. Finally, the quantification of aspartic acid racemization in eye proteins has importance in forensic science and can be used as a measure of chronological aging in humans and other organisms [74,75].

### 4.3. Chronic Kidney Disease

The presence of D-amino acids was intermittently reported in patients with kidney diseases, though recent studies unraveled the clinical significance of D-amino acids in chronic kidney diseases (CKD) [40,76]. CKD is well recognized globally as a significant clinical and public health problem and it is estimated to affect almost one billion humans [41]. The common pathological alteration in progressive CKD is tubulointerstitial fibrosis, which involves various independent and overlapping cellular and molecular pathways leading to reduction in the glomerular filtration ratio (GFR). The disease has high morbidity and mortality ratios, and end-stage patients need careful monitoring of diet, as dietary contents and their metabolites are linked to the disease progression [77,78]. A core issue in the management of CKD is the early diagnosis of kidney diseases. In this regard, the levels of some D-amino acids are identified to be in correlation with GFR. Indeed, the plasma D-Ser level was associated with creatinine-based estimated GFR, and this finding was thought to offer new biomarkers to reflect kidney function as the currently available kidney markers are affected by other factors such as muscular mass [76,79,80]. Notably, D-Ser is not the only D-amino acid that is associated with kidney diseases, indeed, D-Asn, D-Ala and D-Pro all have significant levels in patients with kidney diseases in comparison with healthy people. In essence, the levels of D-Pro and D-Asn were found to have a significant correlation with kidney function (estimated GFR). Moreover, the levels of D-Ala and D-Pro correlated well with patients age, while D-Asp and D-Pro were associated with co-presence of diabetes [40]. Recently, researchers successfully developed a three-dimensional HPLC method to detect the trace amounts of D-amino acids in patients with CKD and the correlation with GFR. Good association was determined between kidney function and the levels of D-Ser and D-Asn in particular [41].

### 4.4. Cancer

The possible correlation between D-amino acids and cancer was controversial for a period of time. The first report for the potential link between D-amino acids and cancer was published almost 80 years ago by Kögl and Erxleben. They reported the detection of some D-amino acids, in particular D-Glu, in tumor cell proteins, and concluded that the tumor development was dependent on the presence of these D-amino acids [81]. However, these findings were contraindicated later by many researchers as summarized in this review [82]. It is worthy to note the technical difficulties at that time to detect the presence of trace amounts of D-amino acids. This controversial argument continued for a while as some reports revealed insignificant differences of D-amino acids levels between healthy patients and patients with tumors, while others concluded no differences [83,84]. In gastric cancer patients who are also helicobacter pylori-positive there were high levels of D-Ala in their gastric juice, and this observation influenced other researchers to develop a non-invasive test to detect the presence of these D-amino acids in saliva and enhance the early detection of gastric cancer [84,85]. In contrast, the levels of D-Glu and D-Gln were lower in patients with hepatocellular carcinoma compared to healthy individuals [86]. One can conclude that D-amino acids levels could be different from one tumor condition to the other and there is no universal assumption. Truly, the altered amino acid profiles, particularly D-amino acids, may have great potential in the early detection of some tumors and could be recognized as potential oncometabolites [87,88,89]. Indeed, the levels of D-Ser and D-Asp were significantly higher in MCF-7 cancer cells compared to non-tumorigenic MCF-10A epithelial cells. The elevation of these D-amino acids could be a result of the upregulation of the enzyme racemases. These findings suggest possible roles of these D-amino acids in breast cancer proliferation and thus consider them as potential oncometabolites for breast cancer [87]. Although only D-Ser racemase has been identified in humans and responsible for D-Ser formation, a recent report suggests a role of this enzyme in the formation of D-Asp as well [90]. The reader is advised to a recently published excellent review on the possible roles of D-amino acids as cancer biomarkers and treatment targets [91].

## 5. Recent Advancement in Separation Techniques and the Impact on the Detections of D-Amino Acids and DAACPs

**Figure 2:**
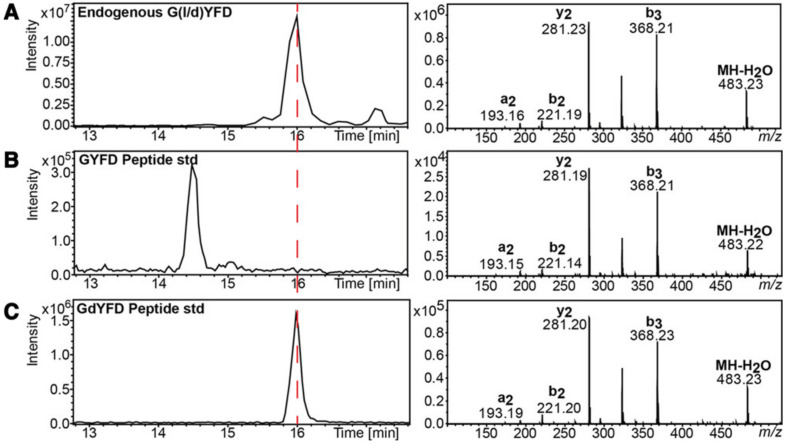
LC–MS analysis of DAACP isolated from *Aplysia californica*, the retention times of the indigenous (**A**) and synthetic DAACP (**C**) are identical and are different from the peptides with all L-amino acids (**B**). However, the MS profiles of the three peptides are similar. Reproduced with permission from [94].

Generally speaking, D-amino acids and DAACPs are present in trace amounts in living organisms, which makes their identification and quantification a challenging task. In multi-omics approaches such as proteomics and metabolomics, mass spectrometry (MS) plays a core role in the analysis of different proteins and peptides. MS analysis requires only limited amounts of the analytes, and it can also be coupled online with separation techniques such as liquid chromatography (LC) and capillary electrophoresis (CE). The availability of deconvolution software and improvements in bioinformatics made MS the main analytical technique for peptide sequencing and protein analysis, particularly when dealing with samples in complex mixtures. Truly, MS techniques were successful in determining many PTMs in proteins such as phosphorylation, acetylation and others [92,93]. However, the modification of amino acids in a peptide to the D-form will not result in a change in the *m/z* values, which challenges the MS detection of such modifications. Nonetheless, MS is capable of detecting the presence of these peptides, albeit with some difficulties. The physicochemical properties of L/D-forms of an amino acid are similar, it is only their reflection of the polarized light which is different. Therefore, in order to separate them, for example by LC, a derivatization step is first required with some chiral molecules and the detection with chiral chromatography. In contrast, diastereomeric peptides, where the chirality of one (or more) amino acid is inverted, have different physicochemical properties which permit their separation by LC without being derivatized. However, the fragmentation pattern in MS will likely be similar, and as a result the differentiation will be challenging. In fact, the Sweedler group proposed a detailed approach for the untargeted discovery of DAACPs, combining enzymatic screening, chiral amino acid analysis and LC–MS. By using this protocol, they were able to detect the presence of a novel bioactive DAACP in *Aplysia californica*. The discovered DAACP has a similar MS profile as a peptide with no D-amino acid in its backbone (Figure 2), and they confirmed the presence of this DAACP by comparing the retention time with a synthetic DAACP [94,95].

**Figure 3:**
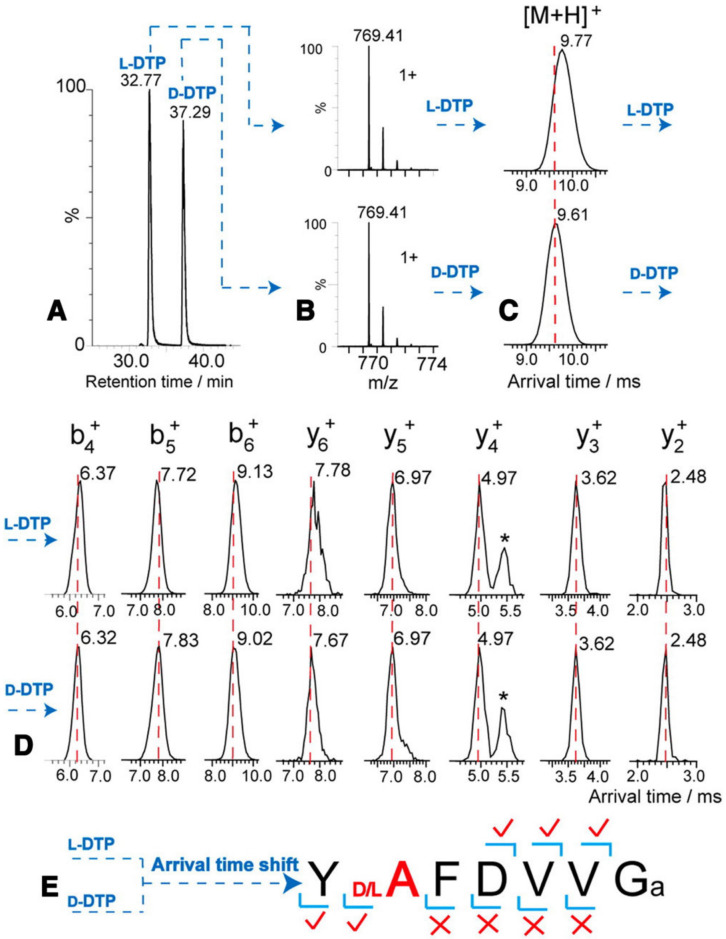
Identification of the epimerization site with LC–MS IMS by comparing the arrival times of the ions of deltorphin having L-Ala (labeled L-DTP) or D-Ala (labeled D-DTP). (**A**,**B**) LC–MS separation shows similar MS profiles and different retention times, indicating that they are isomeric peptides. (**C**) A shift in arrival time is detected for the L/D-forms. (**D**,**E**) Following IMS fragmentation, the arrival times of y_2_^+^, y_3_^+^, y_4_^+^ and y_5_^+^ are similar in both L-DTP and D-DTP, while the arrival times of the y_6_^+^ ions are different, therefore determining the epimerization site. *, interference ions. Reproduced with permission from [107].

Consequently, the presence of indigenous DAACPs could be missed if not compared with the previously synthesized one. Therefore, unless one has the synthetic peptides to compare the retention times with, one might miss the detection of the indigenous DAACPs. This raises a question of how many DAACPs have not been detected and instead were recognized as their all L-amino acids counterparts. What is more difficult is pointing to the specific site of the isomerization, as it is not practical to synthesize all possible DAACPs to compare the retention times. However, some fragmentation techniques such as electron-capture dissociation (ECD) and electron-transfer dissociation (ETD) produce unique fragmentation patterns when α-Asp or β-Asp is present [96,97,98]. Additionally, radical-directed dissociation (RDD) fragmentation was successful in discriminating DAACPs epimers as it is sensitive to the structure of the ions being fragmented. Certainly, RDD fragmentation was better than collision-induced dissociation (CID) in discriminating biologically-active peptides containing D-amino acids [99]. A comprehensive analysis protocol for differentiation of peptide isomers and epimers of lens crystallin using RDD was recently published [100]. Furthermore, the presence of the four Asp isomers in protein and peptides was identified using LC–MS, and three commercial enzymes without the need for synthesized reference peptides. The protein was first hydrolyzed by trypsin, then the tryptic peptides were further hydrolyzed by either Asp-N, Protein L-isoaspartyl methyltransferase (PIMT), or paenidase. These three enzymes hydrolyze the peptides that have L-α-Asp, L-β-Asp and D-α-Asp, respectively. Indeed, this method succeeded to identify all Asp isomers in tryptic peptides of aged lens proteins [43,101]. Notably, the presence of a D-amino acid in a peptide can resist the hydrolysis by certain proteases, and this fact was used to detect the presence of DAACPs. One of the enzymes used for this purpose is Aminopeptidase M (AMP) which hydrolyzes the peptide from the N-terminus. However, AMP digestion stops because of steric hinderance when it reaches a D-amino acid, and in fact some peptides can resist AMP digestion for several days. Nonetheless, the presence of Proline also resists AMP digestion, which will produce false positive results [65,94]. The current advancements in ion mobility spectrometry (IMS) make it an excellent choice for isomeric biomolecules separation [102]. Despite that the theory and principles of IMS were stablished several decades ago, fundamental contributions only started very recently in omics sciences [103]. Moreover, as the coupling of IMS and MS will provide an additional separation dimension, this will allow the detection of isomeric and isobaric molecules in complex mixtures. Eventually, this will lead to greater understanding and characterization of the properties of biomolecules such as proteins and metabolites [104,105]. IMS measures the arrival times of ions in the gaseous phase under applied electric field and vacuum or atmospheric pressure. Ion mobility under these conditions is affected by size, charge and shape of the ions. Consequently, this technique separates molecules in the gaseous phase based on the differences of their mass to charge and shape [104]. Hence, the arrangement of the functional groups is different in DAACPs; therefore, this will change the cross-sectional area of the molecule, and as a result the arrival time of ions will be changed. Subsequently, IMS will permit the direct determination of the site where isomerization takes place (Figure 3). Indeed, a recently published method was successful in discriminating isomeric peptides and instantaneously pointed to the site of epimerization by combining LC–MS and IMS [106,107].

Additionally, a recently developed IMS method was able to baseline separate four Aβ17-28 tryptic peptide epimers on a rapid time scale [108]. In this study, sodium adduct ions, [M + H + Na]^2+^, allowed the complete separation of all Aβ epimer sets assessed, which were unachievable for their [M + 2H]^2+^ doubly protonated ions. IMS coupled with MS was also applied for the detection of free D-amino acids after derivatization with a chiral reagent to diastereomers. This novel analytical method was able to detect D-amino acids in the nanomolar range, and the analysis time including the derivatization step was less than 15 min [109]. Notably, several chiral derivatization reagents were used for the chromatographic separations of D-amino acids; however, Marfey’s reagent is the mostly used one [110,111,112,113]. Recently, a pair of stereodynamic chiral benzylicaldehyde probes were developed for the determination of amino acid configuration in peptides by MS. This method was accurate and the obtained results were well correlated when compared with Marfey’s derivatization methods, without the need for tedious and time-consuming separation steps [114]. It is worth mentioning that not only the analytical techniques are used for the detection and quantification of D-amino acids. Additionally, there are reported protocols for the detection of D-amino acids by enzymatic assay methods, and the most exploited enzyme in these assays is D-amino oxidase. These enzymatic assays provide the advantages of high sensitivity and specificity which allow the development of low-cost protocols and avoid the tedious and time-consuming steps in the analytical techniques [115]. Moreover, there is ongoing research to develop enzymatic biosensors for the rapid detection of D-amino acids [116]. Eventually, several biosensors were fabricated for the detection of D-amino acids in biological samples, and they showed rapid response, good sensitivity and high recovery of the added D-amino acids [117,118]. Pundir et al. and Rosini et al. published excellent reviews on the use of biosensors for the detection of D-amino acids [116,119]. Similarly, for more deep details in the recent progress of the approaches for the detection of isomerism in peptides and proteins, see the review written by Erik T. Jansson [120]. In addition, the research on detecting these potential disease biomarkers is ongoing, and more lab-on-chip devices were developed or are on the road for disease detection [119,121].

## 6. Conclusions

The tertiary structure of proteins is crucial for their functions and activity, in the way that minor changes in the amino acid sequence may lead to significant influence on the activity. Homochirality in protein synthesis is one of life’s mysteries, yet the presence of DAACPs in different tissues represent a new insight to unravel several molecular mechanisms in these tissues. The levels of some free D-amino acids in body are found to be linked with many diseases. Consequently, the presence of DAACPs and D-amino acids in significant levels in some disease conditions will make them potential biomarkers and therapeutic targets for those diseases. In the human body, most cells are replaced after a certain period of time; however, some cells of relatively long life spans, such as neurons, are more likely to accumulate more DAACPs. Though DAACPs and D-amino acids are present in trace amounts, the recent advances in analytical techniques permit more accurate analysis of them. Subsequently, this will assist in better understanding on DAACPs and D-amino acids and unravel their association with disease development and progress.

## Data Availability

Not applicable.
